# Elacestrant Improves Progression-Free Survival After Endocrine Therapy for Estrogen Receptor-Positive Metastatic Breast Cancer

**DOI:** 10.1093/oncolo/oyac015

**Published:** 2022-03-28

**Authors:** Anne Jacobson

## Abstract

In the phase III EMERALD trial, treatment with elacestrant significantly improved progression-free survival compared with investigator’s choice in patients with estrogen receptor-positive metastatic breast cancer that progressed on prior endocrine therapies.

Endocrine therapy in combination with a cyclin-dependent kinase 4/6 (CDK4/6) inhibitor has emerged as the standard of care for patients with estrogen receptor (ER)-positive, human epidermal growth factor receptor 2 (HER2)-negative metastatic breast cancer. However, most patients will experience disease progression on first-line therapy related to the development of treatment resistance, including resistance secondary to the development of *ESR1* mutations.

After progression on first-line therapy, options for patients with ER-positive/HER2-negative metastatic breast cancer include sequential endocrine therapy, with the goal of exhausting available endocrine therapies before switching to chemotherapy. Standard single-agent endocrine therapies such as fulvestrant are associated with poor progression-free survival (PFS), averaging approximately 2 months in the second- and third-line settings.

Elacestrant is an investigational, oral, selective estrogen receptor degrader (SERD) that demonstrates antitumor activity in ER-positive/HER2-negative metastatic breast cancer previously treated with fulvestrant and CDK4/6 inhibition.^1^ Elacestrant also shows activity in tumors harboring *ESR1* mutations.^1^ The phase III EMERALD trial was designed to evaluate elacestrant in patients with estrogen receptor (ER)-positive/HER2-negative metastatic breast cancers that progressed on prior treatment with endocrine and targeted therapies.

Aditya Bardia, M.D., M.P.H., of Mass General Cancer Center, presented results from the EMERALD trial, the first phase III study to examine an oral SERD in advanced breast cancer.^2^

## Study Design

The EMERALD trial enrolled 477 men and postmenopausal women with ER-positive/HER2-negative metastatic breast cancer who had received 1-2 prior lines of endocrine therapy for advanced disease. Up to 1 prior line of chemotherapy in the advanced setting was also permitted. All patients progressed or relapsed on or after prior endocrine therapy given alone or in combination with a CDK4/6 inhibitor.

Patients were randomly assigned to treatment with elacestrant or standard of care, defined as the investigator’s choice of fulvestrant or an aromatase inhibitor. The co-primary endpoints were PFS in all patients and PFS in patients with tumors harboring *ESR1* mutations.

Baseline characteristics were similar in both treatment groups (Table 1). At enrollment, 115 patients in the elacestrant arm and 113 patients in the standard-of-care arm had tumors with mutated *ESR1*.

**Table 1.** Baseline characteristics of patients with ER-positive metastatic breast cancer

Characteristic	Elacestrant (*n* = 239)	Standard of Care (*n* = 238)
Median age	63.0 years	63.5 years
Female patients	97.5%	99.6%
ECOG performance status		
0	59.8%	56.7%
1	40.2%	42.9%
>1	0%	0.4%
Visceral metastasis	68.2%	70.6%
Bone-only disease	15.9%	12.2%
Prior adjuvant therapy	66.1%	59.2%
Number of prior lines of endocrine therapy		
1	54.0%	59.2%
2	46.0%	40.8%
Number of prior lines of chemotherapy		
0	79.9%	75.6%
1	20.1%	24.4%

## Key Findings

Elacestrant significantly reduced the risk of disease progression or death compared with standard endocrine therapy (Table 2). In the overall study population, the median PFS was 2.79 months in the elacestrant group and 1.91 months in the standard of care group. This represents a 30% reduction in the risk of progression or death with the oral SERD (HR, 0.69; *p* =.0018).

**Table 2.** Progression-free survival in patients with ER-positive metastatic breast cancer

Endpoint	Elacestrant	Standard of Care	HR (95% CI)	*p* value
All patients	(*n* = 239)	(*n* = 238)		
Median PFS	2.79 months	1.91 months	0.697 (0.552-0.880)	.0018
Patients with *mESR1*-positive tumors	(*n* = 115)	(*n* = 113)		
Median PFS	3.78 months	1.87 months	0.546 (0.387-0.768)	.0005

Among patients with tumors harboring *mESR1*, elacestrant was associated with a 45% reduction in the risk of progression or death compared with standard therapy. In this subgroup, the median PFS was 3.79 months with elacestrant and 1.87 months with standard endocrine therapy (HR, 0.54; *p* =.0005).

Elacestrant was associated with a higher PFS compared with standard therapy at 6 months (34.3% vs. 20.4%) and 12 months (22.3% vs. 9.4%), suggesting a sustained benefit with oral SERD treatment. The observation of higher PFS was consistent for patients with tumors harboring *mESR1*. In this subgroup, elacestrant demonstrated a higher PFS rate compared with standard therapy at 6 months (40.8% vs. 19.1%) and at 12 months (26.8% vs. 8.2%).

In the safety analysis, elacestrant demonstrated a predictable safety profile consistent with that of other endocrine therapies (Table 3). The most common adverse events in the elacestrant arm included nausea, fatigue, vomiting, decreased appetite, and arthralgia. Adverse events leading to treatment discontinuations were infrequent in the elacestrant and standard-of-care groups (6.3% and 4.4%, respectively).

**Table 3.** Adverse events with elacestrant and standard endocrine therapy

Adverse event	Elacestrant (*n* = 237)		Standard of Care (*n* = 229)	
	All grades	Grade 3-4	All grades	Grade 3-4
Nausea	35.0%	2.5%	18.8%	0.9%
Fatigue	19.0%	0.8%	18.8%	0.9%
Vomiting	19.0%	0.8%	8.3%	0%
Decreased appetite	14.8%	0.8%	9.2%	0.4%
Arthralgia	14.3%	0.8%	16.2%	0%
Diarrhea	13.9%	0%	10.0%	0.9%
Back pain	13.9%	2.5%	9.6%	0.4%
Elevated aspartate aminotransferase	13.1%	1.7%	12.2%	0.9%
Headache	12.2%	1.7%	12.2%	0.9%
Constipation	12.2%	0%	6.6%	0%
Hot flush	11.4%	0%	2.6%	0%
Elevated alanine aminotransferase	9.3%	2.1%	10.0%	0.4%

In summary, elacestrant is the first oral SERD to demonstrate a significant improvement in PFS in a phase III trial of patients with ER-positive/HER2-negative metastatic breast cancer, suggesting a potential role for this novel therapy in the second- and third-line treatment settings.

## Notes

Please note that this content was created for the purpose of summarizing the content of the San Antonio Breast Cancer Symposium 2021 and has not been peer reviewed under the standard policies of *The Oncologist*.
